# Effects of Different Treatment of Fecal Microbiota Transplantation Techniques on Treatment of Ulcerative Colitis in Rats

**DOI:** 10.3389/fmicb.2021.683234

**Published:** 2021-07-14

**Authors:** Fangyuan Zhu, Yifan Ke, Yiting Luo, Jiaqian Wu, Pei Wu, Fangxiao Ma, Yingchao Liu

**Affiliations:** ^1^The 2nd Clinical Medical College, Zhejiang Chinese Medical University, Hangzhou, China; ^2^Academic Affairs Office, Zhejiang Chinese Medical University, Hangzhou, China

**Keywords:** frozen fecal microbiota transplantation, fresh fecal microbiota transplantation, ulcerative colitis, intestinal microbiota, donor

## Abstract

**Background:** Ulcerative colitis (UC) is a chronic non-specific inflammatory bowel disease with abdominal pain, mucus, pus and blood in the stool as the main clinical manifestations. The pathogenesis of UC is still not completely clear, and multiple factors, such as genetic susceptibility, immune response, intestinal microecological changes and environmental factors, together lead to the onset of UC. In recent years, the role of intestinal microbiota disturbances on the pathogenesis of UC has received widespread attention. Therefore, fecal microbiota transplantation (FMT), which changes the intestinal microecological environment of UC patients by transplantation of normal fecal bacteria, has attracted increasing attention from researchers. However, there are no guidelines to recommend fresh FMT or frozen FMT in the treatment of UC, and there are few studies on this. Therefore, the purpose of this study was to explore the effects of fresh and frozen FMT methods on the treatment of experimental UC models in rats.

**Results:** Compared with the model control group, all FMT groups achieved better efficacy, mainly manifested as weight gain by the rats, improvements in fecal characteristics and blood stools, reduced inflammatory factors and normal bacterial microbiota. The efficacy of the frozen FMT group was better than that of the fresh FMT group in terms of behavior and colon length.

**Conclusion:** FMT method supplements the gut microbiota with beneficial bacteria, such as short-chain fatty acid-producing bacteria. These bacteria can regulate intestinal function, protect the mucosal barrier and reduce harmful bacteria, thus mitigating the damage to the intestinal barrier and the associated inflammatory response, resulting in UC remission. FMT is a feasible method for treating UC, with frozen FMT having a superior therapeutic effect than that of fresh FMT.

## Introduction

Ulcerative colitis (UC), a chronic non-specific inflammatory bowel disease that occurs mostly in young adults, affects the colorectal mucosa. The primary clinical manifestations are abdominal pain, mucus, pus and blood in the stool (Ordás et al., 2012; Eisenstein, 2018). UC is a long-lasting disease with recurrent attacks, and in severe cases, intestinal perforation, colon cancer and even death may occur. The pathogenesis of UC is not completely clear, and multiple factors, such as genetic susceptibility, immune response, intestinal microecological changes and environmental factors, together lead to the onset of UC (Ungaro et al., 2017). In recent years, the role of intestinal microbiota disturbances on the pathogenesis of UC has received widespread attention, as disruption of the gut bacteria can damage the intestinal mucosa; influence T-cell subgroup differentiation; cause imbalances in T-helper (Th)l, Th2 and Th17, and regulatory T cells; and lead to secretion of a large number of inflammatory mediators, such as interleukin (IL)-1β, IL-5, IL-6, IL-17 and tumor necrosis factor alpha (TNF-α; Danese and Fiocchi, 2011; Ungaro et al., 2017). Additionally, changes in intestinal immune functions can occur, leading to UC and potentially to further complications. Changes in various intestinal microbiota genera play a key role in the incidence of UC (Kaakoush et al., 2014; Paramsothy et al., 2019). Thus, maintaining the balance of the intestinal microbiota is essential for alleviating the symptoms of UC. Therefore, fecal microbiota transplantation (FMT), which changes the intestinal microecological environment of patients with UC *via* transplantation of normal fecal bacteria, has attracted increasing attention (Vindigni and Surawicz, 2017; Weingarden and Vaughn, 2017).

At present, FMT is mainly used to treat diseases, such as *Clostridium difficile* infection-associated diarrhea (van Nood et al., 2013; Kelly et al., 2016), inflammatory bowel disease (Weingarden and Vaughn, 2017; Shen et al., 2018), irritable bowel syndrome (El-Salhy et al., 2019; Johnsen et al., 2020) and non-alcoholic fatty liver (Rossen et al., 2015b; Craven et al., 2020). FMT has a clear effect on recurrent *C. difficile* infectious diarrhea and is recommended by the European Society of Microbiology (Cammarota et al., 2017). The Joint Guidelines of the British Society of Gastroenterology and the Medical Infectious Society recommend that frozen FMT materials used to treat *C. difficile* infection should be considered as superior to fresh FMT preparations (level of evidence: high; recommended strength: strong; Mullish et al., 2018). However, there are no guidelines for recommending fresh FMT or frozen FMT in the treatment of UC, and these treatments have not been widely examined. Therefore, we compared the efficacity of fresh and frozen FMT methods in treating UC in rats.

## Materials and Methods

### Animals and Groups

Forty specific pathogen-free-grade male Sprague–Dawley rats weighing 240–250 g were purchased from Shanghai SLAC Laboratory Animal Co., Ltd. [Experimental Animal Production License Number: SCXK (Shanghai) 2017-0005]. The rats were raised in the Animal Experiment Center of Zhejiang Chinese Medical University [Experimental Animal License Number: SYXK (Zhejiang) 2018-0012], which provided a barrier environment. During the feeding period, animals were given standard pellet feed and free drinking water and housed at a controlled temperature (23 ± 2°C), humidity (60%) and a 12 h light cycle. Animal handling during the experiment complied with the ‘Guiding Opinions on the Good Treatment of Laboratory Animals’ issued by the Ministry of Science and Technology Under Government of China in 2006. The study was approved by the Ethics Committee of Zhejiang Chinese Medical University (IACUC-20200506-12).

According to the random number table method, the rats were randomly divided into two groups: a control (non-UC) group with eight animals and UC model group with 32 animals. After both groups were subjected to 1 week of adaptive feeding, the UC model group was created. The rats were anesthetized with 3% pentobarbital sodium (0.15 ml/100 g). A rubber infusion tube was then inserted 8 cm into the upper anus of each rat and injected with 5% 2,4,6-trinitrobenzene sulfonic (TNBS) acid at 100 mg/kg (dissolved in 50% ethanol, total volume of 1 ml; Liu et al., 2016; Silva et al., 2019). To prevent fluid leakage after colonic infusion and ensure the induction of colitis, the rats were placed in the Trendelenburg position with their head down for 1 min (Rashidian et al., 2016). The daily weight and disease activity index (DAI) score of both control and UC model groups of rats were recorded. Fresh feces were collected from each group before and after UC model group creation. After the experiment, the rats were killed by CO_2_ inhalation, and colon tissue specimens and feces were collected and immediately placed at −80°C.

The UC model group was randomly divided into four groups 72 h later, with eight animals in each group: UC model group (control), mesalazine group, fresh FMT group and frozen FMT group. The rats in each group were fed separately in cages.

### Main Reagents and Instruments

Trinitrobenzene sulfonic (lot: SLCD2161) was purchased from Sigma (St. Louis, MO, United States). Mesalazine (lot: 14055) was purchased from Shanghai Haoyuan Chemexpress Co., Ltd (Shanghai, China). The rat TNF-α ELISA Kit (lot: 20200930) was purchased from Nanjing Jiancheng Bioengineering Institute (Nanjing, China). A BioTek luciferase chemiluminescence detector (Winooski, VT, United States) was also used.

### Interventions

#### Fecal Bacteria Preparation

For frozen FMT preparation, 7 days before transplantation, the control group was treated as donors whose fecal samples were collected in a sterile tube, dissolved in 0.9% NaCl at a ratio of 1 g:10 ml and homogenized for 5 min, followed by adding 0.1 ml 10% glycerol, mixing and freezing at −80°C (Hamilton et al., 2012). The samples were transferred to a −80°C refrigerator for 1 week.

For fresh FMT preparation, on transplant day, the control group was treated as donors whose samples were collected in a sterile tube, dissolved in 0.9% NaCl at a ratio of 1 g:10 ml and homogenized for 5 min, followed by 0.1 ml of 10% glycerol. The time from sampling to successful transplantation did not exceed 3 h (Smits et al., 2013).

#### Intervention Pattern of Each Group

Rats were randomly divided into four groups (*n* = 8, per group). The rats were administered different treatments once per day for five consecutive days. The fresh FMT group was treated with 1 ml/100 g of fresh fecal homogenate from the control group (non-UC) on the same day of transplantation, whereas the frozen FMT group was treated with 1 ml/100 g of frozen fecal homogenate collected from the control group 7 days prior to transplantation. The mesalazine group was administered 0.3 g/kg of mesalazine by gavage as a positive control. The UC model group and control group were administered 1 ml/100 g of saline by gavage as a blank control and negative control, respectively.

### Specimen Collection

The feces of the rats were collected at 72 h after model group creation, 2 days after stopping drug intervention and 7 days after stopping drug intervention, and immediately placed in −80°C until analysis. After stopping the drug intervention for 7 days, the rats were killed by CO_2_ inhalation. The colon tissue was separated, and the most severely affected part of the colon tissue assessed visually; excess fat around the tissue was cut-off. The tissues were repeatedly rinsed with pre-cooled normal saline, after which filter paper was used to absorb the excess liquid, and the colon tissue was placed in 4% polyoxymethylene for fixing (lot: 69111800, Biosharp) and preserved in formaldehyde solution until hematoxylin-eosin staining. A section of severely diseased colon tissue was placed in a sterile cryopreservation tube and transferred to −80°C for subsequent detection of inflammatory factors.

### Detection Indicator

#### Behavioral Score

Weight was measured once every day. After model group creation, weight loss, stool traits, blood and stool of each group were observed daily to determine the DAI score, as given in Table 1 (Wirtz et al., 2017).

**Table 1:** Disease activity index score as the sum of weight loss, stool consistency and occult blood or gross blood in the stool.

Score	Weight loss	Stool consistency	Blood
0	None	Normal	None
1	1–5%	Soft but still formed	
2	5–10%	Soft	Positive haemoccult
3	10–15%	Very soft; wet	
4	>15%	Watery diarrhea	Blood traces in stool visible

#### Histological Analysis

Colon tissues fixed with polyoxymethylene solution were embedded in paraffin. After hematoxylin-eosin staining, the tissue structure was observed in detail under a light microscope, and the pathological score was determined according to Table 2 (Wirtz et al., 2017; Wang et al., 2019).

**Table 2:** Pathological score as the sum of the score of infiltration of inflammatory cells in the lamina propria, colonic tissue damage and crypt damage.

Score	Infiltration of inflammatory cells in lamina propria	Colonic tissue damage	Crypt damage
0	Infrequent	None	None
1	Elevated with some neutrophils	Isolated focal epithelial damage	Loss of 1/3 of the basal layer
2	Submucosal presence of inflammatory cell clusters	Mucosal erosions and ulcerations	Loss of 2/3 of the basal layer
3	Transmural cell infiltration	Extensive damage deep into the bowel wall	Only the surface epithelium is intact
4			Loss of entire crypts and epithelium

#### Enzyme-Linked Immunosorbent Assay

The collected colon samples were allowed to thaw at room temperature and then tested for TNF-α using the TNF-α ELISA Kit (lot: 20200930) according to the kit instructions. The absorbance of samples in each well of the plate was measured with a multifunctional microplate reader at a wavelength of 450 nm.

#### Bacterial DNA Extraction and 16S rRNA Gene Sequencing

Total genomic DNA was extracted from the samples using the CTAB/SDS method. DNA concentration and purity were monitored on 1% agarose gels. According to the concentration, DNA was diluted to 1 ng/μl using sterile water. The primers used were 16S V3-V4: 341F 5'-CCTAYGGGRBGCASCAG-3' and 806R 5'-GGACTACNNGGGTATCTAAT-3'. 16S rRNA genes were tagged with a barcode and used for analysis. All polymerase chain reactions (PCRs) were carried out in 30 μl volume containing 15 μl Phusion^®^ High-Fidelity PCR Master Mix (New England Biolabs, Ipswich, MA, United States), 0.2 μm of forward and reverse primers and approximately 10 ng template DNA. Thermal cycling steps consisted of initial denaturation at 98°C for 1 min, followed by 30 cycles of denaturation at 98°C for 10 s, annealing at 50°C for 30 s and elongation at 72°C for 60 s. The last step was performed at 72°C for 5 min. Equal volumes of 1X loading buffer (containing SYB green) and PCR products were mixed, and the samples were evaluated by 2% agarose gel electrophoresis. Samples showing bands at 400–450 base pairs were further analyzed. PCR products were mixed in equivalent ratios and purified with an AxyPrepDNA Gel Extraction Kit (AXYGEN, Union City, CA, United States). Sequencing libraries were generated using an NEB Next^®^ Ultra^™^ DNA Library Prep Kit for Illumina (New England Biolabs) following the manufacturer’s recommendations, and index codes were added. The library quality was assessed on a Qubit 2.0 Fluorometer (Thermo Fisher Scientific, Waltham, MA, United States) and Agilent Bioanalyzer 2100 system (Agilent Technologies, Santa Clara, CA, United States). The library was sequenced on an Illumina NovaSeq 6000 platform (San Diego, CA, United States), and 250-bp/300-bp paired-end reads were generated.

### Statistical Analysis

The measured data were expressed as the average ± standard deviation (x ± s). SPSS software (version 20.0, SPSS Inc., Chicago, IL, United States) was used to analyze the data. The independent sample *t*-test was used to compared data from the two groups, and one-way analysis of variance was performed to compare the data between multiple groups. *p* < 0.05 indicated statistically significant results. Microbiota data were analyzed and graphed by the non-parametric tests Kruskal-Wallis sum rank test and Wilcoxon rank sum test with R language ggplot2 and ggpubr, and the MetagenomeSeq toolkit and Ubuntu Linux conda LEfSe software.

## Results

### Changes in General Conditions in Each Group

**Figure 1:**
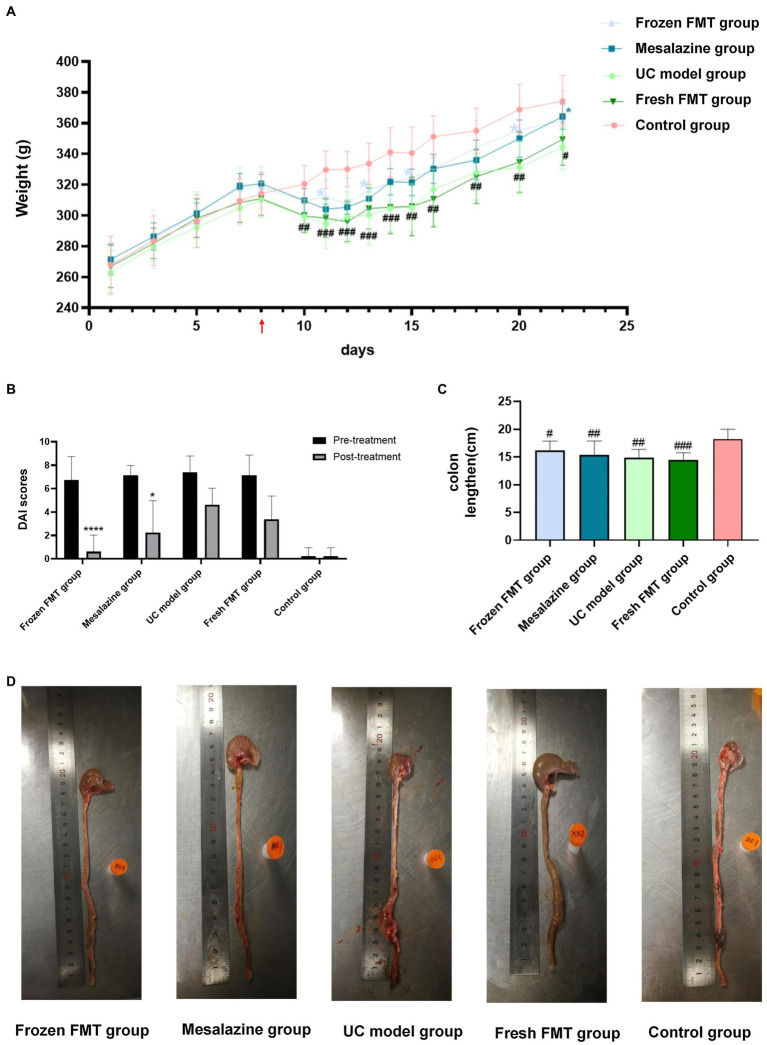
Changes in general conditions of rats in each group. **(A)** Change in mean weight of each group: red arrow in the picture shows the day of model creation. Due to fasting for 24 h before the model creation, no weight was recorded on this day, *n* = 6. **(B)** Disease activity index score: as trinitrobenzene sulfonic-induced colitis tends to heal itself, we compared the UC group on the same day after treatment with the other groups, *n* = 8. Comparison before and after treatment. ^*^p < 0.05 and ^****^p <0.0001. **(C)** Scatter plot of colon length in each group, *n* = 8. **(D)** Typical colon anatomy of each group, *n* = 8. Data are expressed as the mean ± SD. #*p* < 0.05, ##*p* < 0.01 and ###*p* < 0.001 vs. control group. ^*^p < 0.05 vs. UC model group.

To evaluate the effect of FMT on the chronic intestinal inflammation conditions similar to that of human patients with inflammatory bowel disease, experimental enteritis in rats was induced by administering TNBS. After starting TNBS treatment, feces from control rats were repeatedly administered to UC model rats by gavage. Figure 1A shows the curve of the average body weight in each group. Before developing the TNBS-induced UC model group, there was no significant difference between groups (*p* > 0.05). After 72 h of model group creation, the weight of the rats in each model group decreased to a similar extent, with differences observed between the control group and other groups (*p* < 0.05). After intervention, there were significant differences in body weight between the frozen FMT group, mesalazine group and UC model group (*p* < 0.05), whereas there was no significant difference between the UC model group and fresh FMT group. There was no difference among the fresh FMT, frozen FMT and mesalazine groups (*p* > 0.05).

After model development, each group began showing various degrees of malaise, arched back, yellow coat, diarrhea, mucus pus and blood in the stool, accompanied by slow weight gain or even weight loss. After intervention, the activity status, bloody stool and diarrhea improved. The DAI score is shown in Figure 1B.

We also measured the colon length. As shown in Figure 1D, the colons of each group show varying degrees of dilatation, oedema and even bleeding ulcers after model creation. However, dilation and oedema of the colon in the three different intervention groups were not as obvious as those in the model group, and there was no obvious ulcer bleeding. According to the colon length statistics, as shown in Figure 1C, only the control group significantly differed from the other four groups (*p* < 0.05). The remaining four groups showed no significant differences (*p* > 0.05).

### Pathological Changes and Scores of Colonic Tissues in Each Group

**Figure 2:**
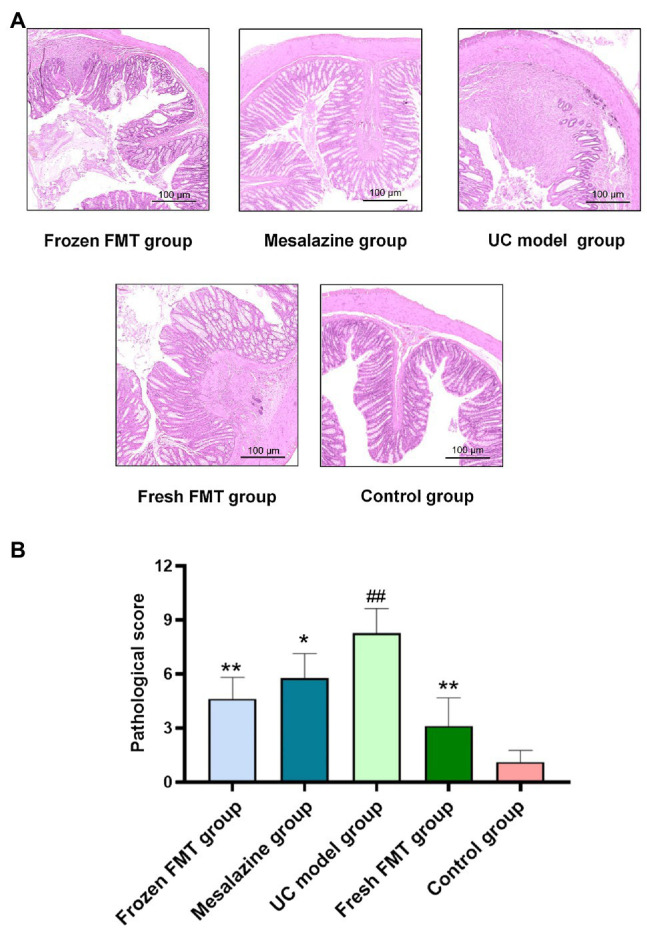
Pathological changes and scores of colonic tissues of rats in each group. **(A)** Typical histological performance of each group. **(B)** Pathological score of each group, *n* = 8. Data are expressed as the mean ± SD. ##*p* < 0.01 vs. control group. ^*^p < 0.05 and ^**^p < 0.01 vs. UC model group.

The typical pathological manifestations and histologic injury scores of the colon in each group are shown in Figure 2. The colon tissue in the control group showed a complete colonic epithelium, regular crypt structure and small amounts of inflammatory cell infiltration. The pathological changes in the UC model group were as follows: obvious erosion and ulceration were observed in the mucosal epithelium; the number and structure of epithelial crypts were changed, and their arrangement was disordered; goblet cells were significantly reduced; high inflammatory cell infiltration was observed in the lamina propria; and a large amount of inflammatory cell infiltration and oedema were observed in the submucosa. However, in pathological sections of the fresh and frozen FMT groups, inflammatory infiltration and destruction of the intestinal wall were milder than those in the UC model group.

### Levels of Inflammatory Factors in Each Group

**Figure 3:**
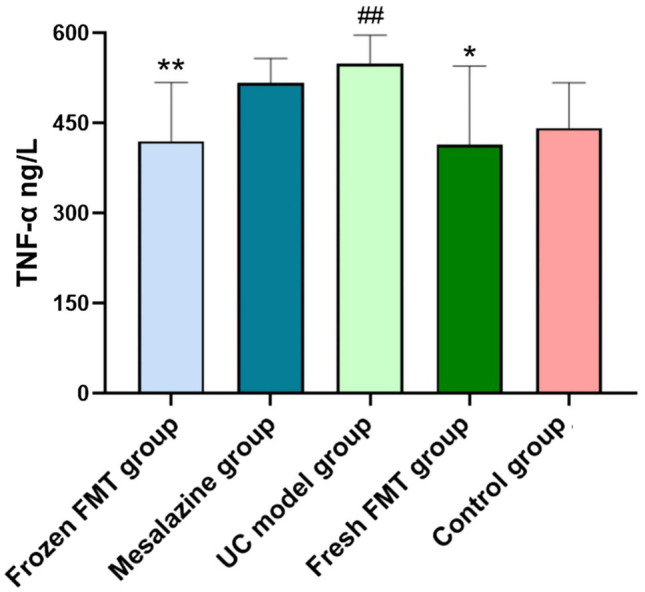
Colon tissue inflammation factor tumor necrosis factor alpha (TNF-α) level in each group, *n* = 8. Data are expressed as the mean ± SD. ##*p* < 0.01 vs. control group. ^*^p < 0.05 and ^**^p < 0.01 vs. UC model group.

The levels of TNF-α in the colon tissues of each group are shown in Figure 3. According to the intervention results of each group, treatment with FMT reduced intestinal inflammation. The expression of TNF-α in the colon of the two FMT groups was significantly lower than that in the model group (*p* < 0.05) and returned to the level in normal rats. There was no significant difference between the fresh and frozen FMT groups (*p* > 0.05).

### FMT Treatment Improves Intestinal Microbiota

The pair-end reads obtained by MiSeq sequencing were first spliced according to the overlap relationship between the sequences, and the sequence quality was controlled and filtered. The samples were distinguished, after which cluster analysis and species taxonomy analysis were performed. Cluster analysis showed that various diversity index analyses could be performed and the depth of sequencing could be detected. Based on taxonomic information, community structure statistics were performed at each classification level.

**Figure 4:**
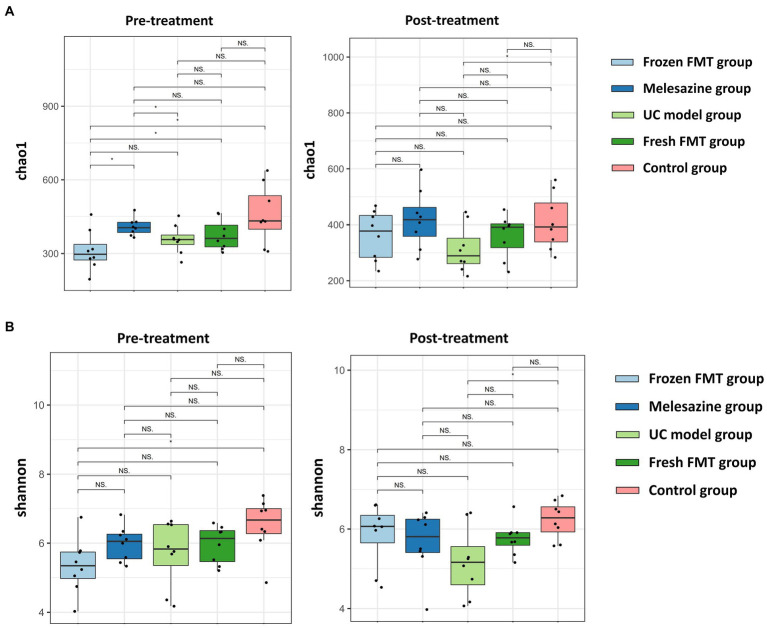
*α* diversity of bacterial groups. **(A)** *α* diversity of each group of microbiota (Chao 1); **(B)** *α* diversity of each group of microbiota (Shannon index). ^*^p < 0.05.

The two factors affecting an alpha diversity index are richness and diversity. We calculated the Chao1 index for the samples to estimate community richness and the Shannon index to estimate community diversity (Figure 4). After model creation, there was a statistical difference in alpha diversity between the frozen FMT group and the control group (*p* < 0.05), but the difference disappeared after intervention (*p* > 0.05), indicating that frozen FMT improved the alpha diversity of the intestinal microbiota in this group. However, this change was not seen in the fresh FMT group.

Each group of samples contained 17 known phyla from the kingdom of bacteria with an abundance of more than 0%, namely Acidobacteria, Actinobacteria, Armatimonadetes, Bacteroidetes, Chloroflexi, Cyanobacteria, Deferribacteres, Elusimicrobia, Epsilonbacteraeota, Firmicutes, Fusobacteria, Gemmatimonadetes, Nitrospirae, Patescibacteria, Proteobacteria, Tenericutes and Verrucomicrobia.

**Figure 5:**
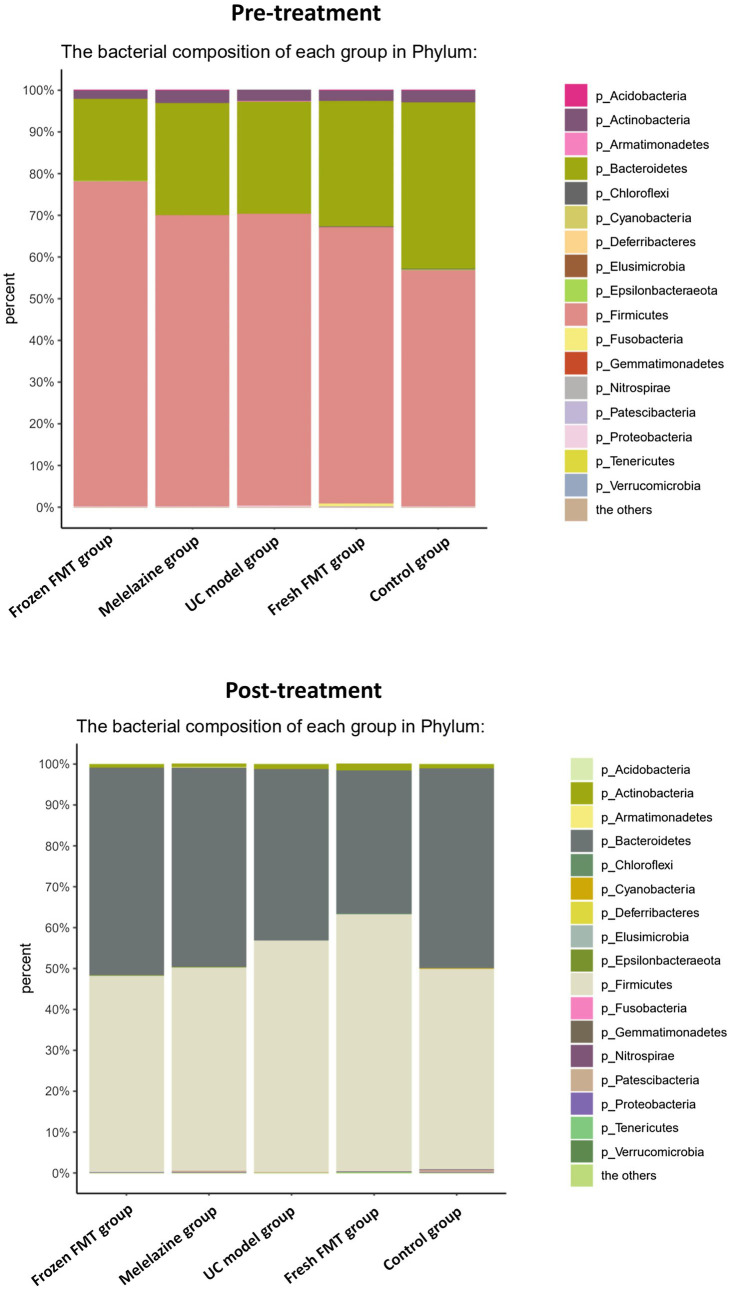
Relative abundance of colonic microbiota composition relative to phylum.

The horizontal analysis of the phylum graph showed that Firmicutes, Bacteroidetes, Actinomycetes and Proteobacteria were predominant (Figure 5). After TNBS-induced induction of UC, the intestinal microbiota of rats differed from that of control rats at the phylum level: the relative abundance of Bacteroidetes was reduced, the relative abundance of Firmicutes was significantly increased, and the relative abundance of Actinomycetes and Proteobacteria was increased. Following intervention, the abundance of the intestinal microbiota of the rats in the frozen FMT and mesalazine groups was significantly changed compared with that following UC model group creation. The relative abundance of Firmicutes, Actinobacteria and Proteobacteria was decreased, whereas that of Bacteroides was increased; significant differences between the frozen FMT group and the UC model group were observed. The fresh FMT group showed a similar trend, yet was not significant from the UC model group.

**Figure 6:**
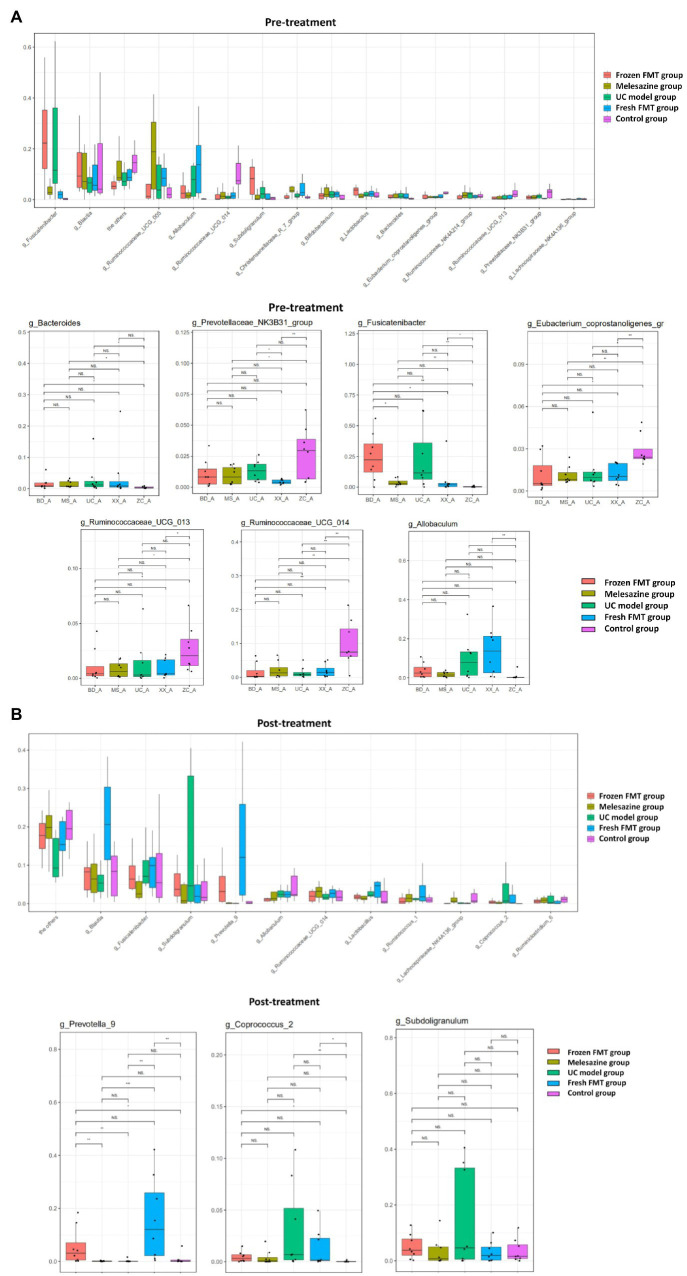
Comparison of species abundance at the genus level. **(A)** Significantly different species in each group before treatment. **(B)** Significantly different species in each group after treatment. ^*^p < 0.05, ^**^p < 0.01, and ^***^p < 0.0001.

At the genus level, the abundance comparison of a single species in each sample group is shown in Figure 6, and the Wilcoxon fit rank test was used to detect differences between groups. This study showed that, in comparison with the control group, the intestinal microbiota of the rats after model creation changes as follows: the abundance of *Bacteroides*, *Christensenellaceae_R_7_group*, *Fusicatenibacter* and *Allobaculum* was increased, whereas *Prevotellaceae_NK3B31_group*, *Ruminococcaceae_UCG_013*, *Ruminococcaceae_UCG_014* and *Eubacterium_coprostanoligenes_group* was decreased. After FMT intervention, the abundance of *Prevotella_9* was increased, and the relative abundance of *Coprococcus* 2 and *Subdoligranulum* was decreased.

**Figure 7:**
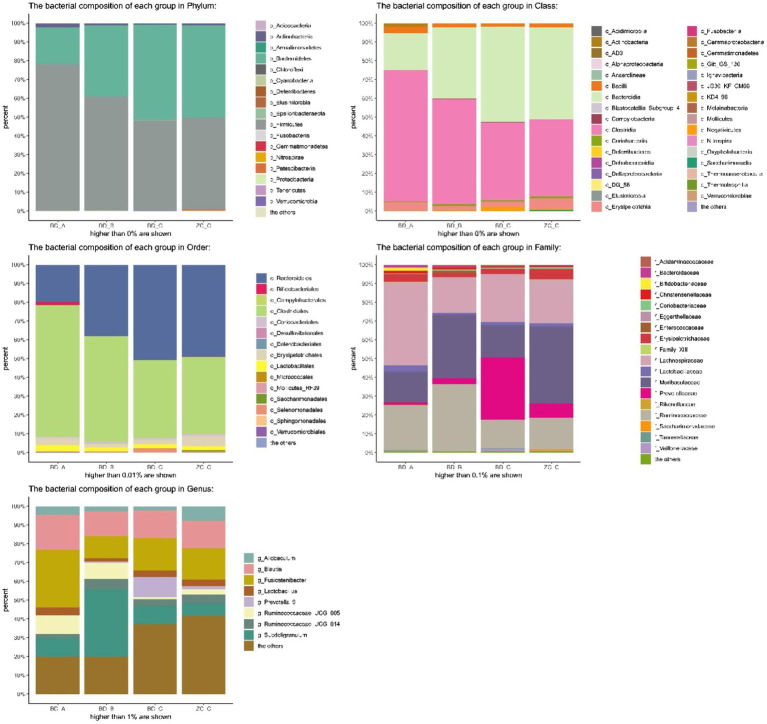
Principal coordinates analysis of frozen fecal microbiota transplantation (FMT) group and fresh FMT group. Frozen FMT group A, frozen FMT group before treatment; Frozen FMT group B, frozen FMT group 1 week after treatment; Frozen FMT group C, frozen FMT group 2 weeks after treatment; Fresh FMT group A, fresh FMT group before treatment; Fresh FMT group B, fresh FMT group 1 week after treatment; Fresh FMT group C, fresh FMT group 2 weeks after treatment.

**Figure 8:**
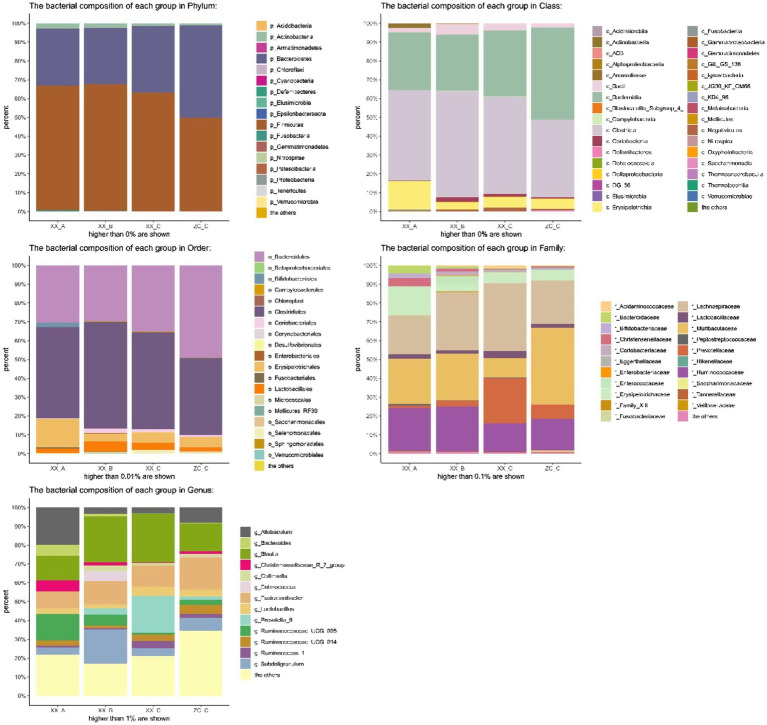
Bacterial community column chart of different groups. BD-A, frozen FMT group before treatment; BD-B, frozen FMT group 1 week after treatment; BD-C, frozen FMT group 2 weeks after treatment; ZC-C, control group; XX-A, fresh FMT group before treatment; XX-B, fresh FMT group 1 week after treatment; XX-C, fresh FMT group 2 weeks after treatment.

Principal coordinates analysis (PCoA) is a non-constrained data dimensionality reduction analysis method used to study the similarities or differences between sample community composition. Different colored points represent samples of different groups; the closer the two sample points are, the more similar the species composition of the two samples. The PCoA data are shown in Figure 7; regardless of whether it was fresh or frozen FMT, the intestinal microbiota after FMT was more similar to the control group compared with that after model creation. In the community column chart shown in Figure 8, whether it was phylum, class, order, family or genus levels, the composition of the colonic microbiota also showed similar results: the colonic microbiota composition of the frozen and fresh FMT groups gradually trended towards that in the control group after intervention. This trend continued to persist after the intervention was terminated.

**Figure 9:**
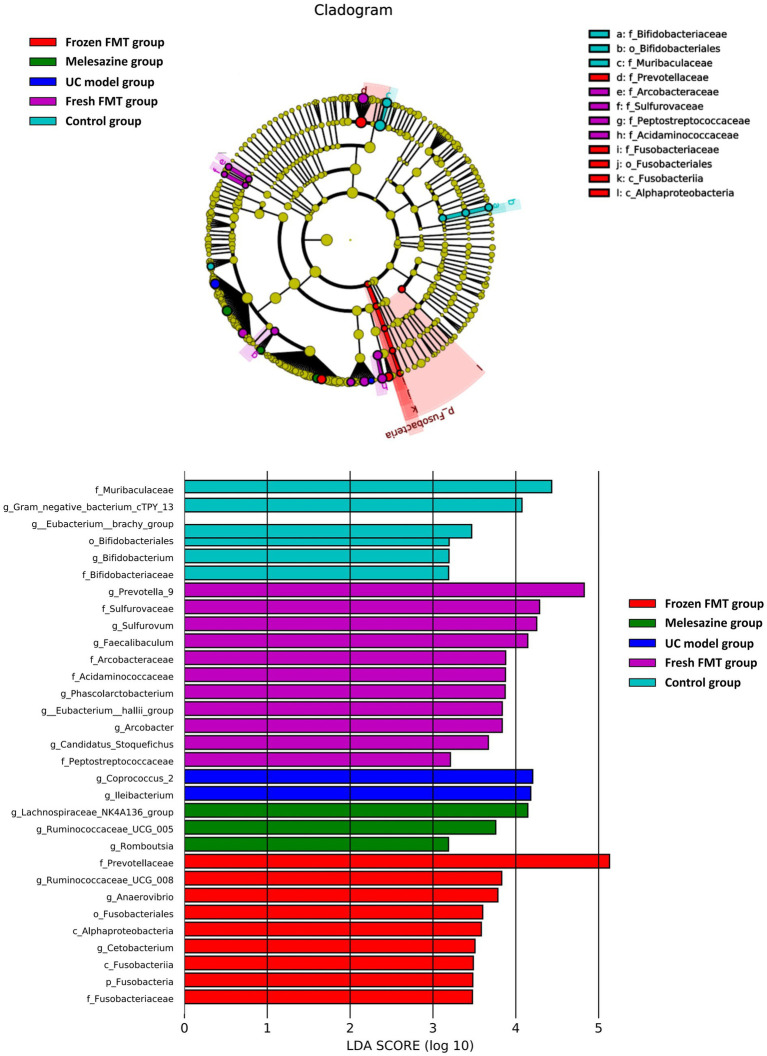
Characteristics of microbial community composition using linear discriminant analysis effect size analysis.

To identify the specific types of bacteria altered by treatment, we performed linear discriminant analysis effect size (LEfSe) analysis to determine the characteristics of different groups of the gut microbiota. The clade map produced by LEfSe analysis revealed species with significant differences in abundance between groups (Figure 9). It shows that the abundance of *Coprococcus 2* and *Ileibacterium* in the UC model group was significantly higher than that in the remaining groups, suggesting that *Coprococcus* 2 and *Ileibacterium* were characteristic bacteria of the UC model group. Similarly, *Fusobacteriaceae*, *c_Alphaproteobacteria*, *Anaerovibrio*, *Ruminococcaceae_UCG_008* and *Prevotellaceae* were characteristic bacteria of the frozen FMT group. *Prevotella_9*, *Acidaminococcaceae*, *Peptostrepto coccaceae*, *Arcobacter*, *Phascolarctobacterium*, *Eubacterium_hallii_group*, *Candidatus Stoquefichus*, *Arcobacteraceae*, *Faecalibaculum* and *Sulfurovaceae* were characteristic bacterial genera of the fresh FMT group. The abundances of *Lachnospiraceae NK4A136*, *Ruminococcaceae_UCG_005* and *Romboutsia* were significantly higher than those in the other groups, which are characteristic bacterial genera of the mesalazine group.

## Discussion

The pathogenesis of UC is complex, and it is currently thought that the interaction between the host and gut microbiota is a key factor. Under normal conditions, innate and acquired immunity in the host tolerates the normal microbiota while preventing the invasion of harmful bacteria. When the balance of intestinal microbiota is disrupted, harmful bacteria in the intestinal tract are rapidly increased, and directly invade and destroy intestinal epithelial cells, leading to immune dysfunction. The release of a large amount of enterotoxin increases the permeability of the intestinal mucosa and damages the intestinal mucosal barrier (Franzosa et al., 2019; Larabi et al., 2019). Intestinal mucosal barrier function decreases, and intestinal microbial microbiota shifts, further destroying the intestinal mucosal barrier, causing a vicious cycle and exacerbating intestinal inflammation.

Fecal microbiota transplantation is the process of transferring fecal bacteria from a donor to a recipient (Rossen et al., 2015b; Malikowski et al., 2017). In recent years, FMT has made great progress in the treatment of UC. It has been reported that patients with UC treated by FMT had a higher disease remission rate than those administered traditional treatment alone (Moayyedi et al., 2015; Rossen et al., 2015a; Costello et al., 2019). Moreover, the diversity of intestinal microbiota in patients treated with FMT was significantly increased compared with that before treatment, and the level of intestinal inflammatory factors decreased (Wei et al., 2018). Fecal bacterial transplantation treatment often varies in the choice of the donor, different treatments of fecal bacteria, transplantation methods and method of the exact guide. We used fresh and frozen FMT in a rat model of UC to explore the differences between the two methods.

Fecal microbiota transplantation was found to improve diarrhea, mucous pus, blood in stool and weight loss in rats. Histologically and pathologically, FMT effectively restored crypt injury, reduced intestinal inflammation and ulcer injury, and restored damaged villi. In addition, FMT effectively downregulated the levels of inflammatory factors TNF-α. Moreover, the structure of intestinal microbiota was further analyzed, which showed that after inoculation with exogenous fecal microbiota, the colonic microbiota composition of rats gradually became similar to that of the control group. This is consistent with the previous studies showing that after FMT, the intestinal microbiota composition of recipients and donors was consistent (Paramsothy et al., 2017; Lleal et al., 2019).

Alpha diversity refers to the diversity in a specific area or ecosystem and is a comprehensive indicator reflecting abundance and uniformity. In this study, the intestinal biodiversity of rats after TNBS-induced UC model decreased. Previous clinical studies have also found that patients with UC also have reduced intestinal biodiversity (Parada Venegas et al., 2019). The richness and diversity of intestinal microbiota of rats after frozen FMT were enhanced; a similar trend was observed in rats after fresh FMT. These findings suggest that FMT could increase gut biodiversity. Following UC model creation, rats developed intestinal microbiota disorders. At the Phylum level, the relative abundance of pathogenic species, such as *Actinomycetes* and *Proteobacteria*, was increased. This is consistent with the previous findings that a relative increase in the levels of *Actinobacteria* and *Proteobacteria* is strongly correlated with inflammatory bowel disease severity (Zhou et al., 2018). Other studies have suggested that Proteobacteria is a microbial signature of dysbiosis in gut microbiota (Shin et al., 2015). After FMT, the abundance of *Actinobacteria* and *Proteobacteria* was significantly reduced, suggesting that FMT could reduce the relative abundance of harmful bacterium. The characteristic bacteria of each group show that the species and abundance of bacteria producing short-chain fatty acids in the rats’ intestines increased after frozen FMT, such as *Anaerovibrio*, Ruminococcaceae and Prevotellaceae. After fresh FMT, the short-chain fatty acid-producing bacteria, such as *Prevotella*, *Eubacterium hallii* and *Faecalibaculum*, were also supplemented. These bacteria are considered as beneficial (Guo et al., 2020). FMT increased the number of beneficial bacteria in the gut and decreased the number of harmful bacteria.

Taken together, these results suggest that FMT can effectively treat UC. FMT may play a role in alleviating UC by adjusting the relative abundance of beneficial and harmful bacteria in the intestine. This is biologically reasonable since, in the early stages of UC, fluctuations in the microbiota are easier to recover (Moayyedi et al., 2015).

However, compared with the fresh FMT group, the frozen FMT group showed a greater relief effect on UC model rats in terms of weight, clinical manifestations and colon length. In terms of histopathology, inflammatory factors in fresh and frozen FMT groups showed improvement in UC model rats, but the difference was not significant. A previous study showed that frozen FMT reduced the number of Gram-negative bacteria in feces, which may explain why frozen fecal transplants are more effective than fresh fecal transplants (Costello et al., 2017). After FMT in this experiment, Gram-negative bacteria, such as *Proteus*, were significantly reduced; however, differences between frozen and fresh FMT observed in this study differed from previous findings.

Whether the microorganisms remain viable over time, particularly the beneficial bacteria, is unclear. Studies have shown that there is no difference in the microbiota of frozen and fresh fecal bacteria at 6 or 7 months. In addition, preservation of frozen bacteria requires further analysis. By studying the aerobic bacterial suspension of 10% glycerol stored at −80°C, Costello et al. (2015) found that aerobes, anaerobes, total coliforms, Bifidobacteria, *Escherichia coli* and Lactobacilli survived for at least 6 months. Different species of bacteria have different sensitivities to freeze–thaw damage. Costello et al. (2015) have shown that *Bifidobacterium* has a better survival rate when refrigerated for 6 months compared to total coliforms and *E. coli*. The difference in the survival rate of different strains in cryopreservation may be clinically important for long-term preservation of FMT, which should be analyzed in further studies.

In summary, we showed that frozen FMT can be used to treat UC, and the curative effect was similar to or even better than that of fresh FMT. Previous studies on *C. difficile* infection have shown that frozen FMT does not significantly affect the implantation and efficacy of microorganisms (Youngster et al., 2014). A random clinical trial showed that the lyophilised product had a slightly lowered efficacy in *C. difficile* infection patients compared with fresh product but resembled other treatments in microbial restoration 1 month after FMT (Jiang et al., 2017). Another clinical randomized controlled trial with a larger sample size showed that among adults with recurrent or refractory C. *difficile* infection (CDI), the use of frozen compared with fresh FMT did not negatively affect the clinical resolution of diarrhea (Lee et al., 2016). A meta-analysis compared fresh and frozen FMT in treatment of recurrent CDI and shows no difference in efficacy between these modes of stool preparation (Quraishi et al., 2017). This is important for practical applications, such as supplier relationships, modes of transport and cost-effectiveness. Compared with fresh FMT, frozen FMT reduces the frequency of donor screening (Lee et al., 2016). This approach can be applied in a wide range of healthcare settings. At present, FMT banks have been established internationally by using the fecal intelligent separation system (Cammarota et al., 2019). China also established the Chinese Fecal Microbiota Transplantation Bank in 2015, aiming to realize the emergency treatment of fecal microbiota in different locations and the standardization of fecal bacteria distribution, preparation and preservation. In addition, collecting frozen stool samples through quarantine and obtaining screening results may ease concerns that fresh FMT could transmit pathogens from donors to recipients.

There were some limitations to our study. First, the experimental period was short. Although the microbiota after FMT tended to be normal, it was not yet stable. The experimental period should be extended to detect the composition of colonic microbiota. Second, only one model was used. Studies have shown that multiple model creation is more similar to the chronic colitis model, and multiple FMT significantly improves the treatment effect. Further studies are required to evaluate this point. Third, the experiments were not duplicated. In future studies, the experimental design will be optimized to increase reproducibility.

## Conclusion

Fecal microbiota transplantation method supplements the gut microbiota with beneficial bacteria, such as short-chain fatty acid-producing bacteria. These bacteria can regulate intestinal function, protect the mucosal barrier and reduce harmful bacteria, thus mitigating the damage to the intestinal barrier and the associated inflammatory response, resulting in UC remission. However, FMT is a feasible method for treating UC, with frozen FMT having a superior therapeutic effect than that of fresh FMT.

## Data Availability Statement

The original contributions presented in the study are publicly available. This data can be found at: NCBI repository, accession number: PRJNA734296.

## Ethics Statement

The animal study was reviewed and approved by the Ethics Committee of Zhejiang Chinese Medical University (IACUC-20200506-12).

## Author Contributions

YK and YL conceived the study and secured the funding. FZ, YL, YK, JW, PW, and FM did experiments and performed the analysis. FZ performed the data analysis and wrote the first draft of the manuscript. All authors read and approved the final manuscript.

### Conflict of Interest

The authors declare that the research was conducted in the absence of any commercial or financial relationships that could be construed as a potential conflict of interest.
